# REFRAMING INTERGENERATIONAL FAMILIAL RELATIONSHIPS THROUGH THE STUDY OF GRANDPARENT-GRANDCHILD CONNECTIONS

**DOI:** 10.1093/geroni/igz038.2333

**Published:** 2019-11-08

**Authors:** Nancy Mendoza, A Nancy Mendoza, Christine A Fruhauf

**Affiliations:** 1 The Ohio State University, Columbus, Ohio, United States; 2 Colorado State University, Fort Collins, Colorado, United States

## Abstract

Intergenerational relationships include non-familial and familial connections. Common familial bonds exist between grandparents and grandchildren. Although grandparent-grandchild connections have over 40 years of research, measurement and design gaps remain. With this paper, we will address new approaches to examining grandparent and grandchild relationships in an effort to understand how this connection impacts our attitudes on aging. Specifically, we will discuss the opportunities of approaching such relationships from a longitudinal perspective. The grandparent-grandchild relationship can span close to 30 years, and yet knowledge of relationship stability and change between individuals in these family roles is limited. We will highlight the conference theme by presenting how social network analysis (SNA) applied to empirical data of grandparents raising grandchildren can reframe aging’s network ties. Further, future research using SNA with grandchildren will be addressed as a way to build on previous work, extending our knowledge of intergenerational relationships from the family perspective.

